# Fluorescent Protein-Based Methods for On-Plate Screening of Gene Insertion

**DOI:** 10.1371/journal.pone.0014274

**Published:** 2010-12-10

**Authors:** Stanley S. C. Wong, Kevin Truong

**Affiliations:** 1 Institute of Biomaterials and Biomedical Engineering, University of Toronto, Toronto, Canada; 2 Edward S. Rogers Sr. Department of Electrical and Computer Engineering, University of Toronto, Toronto, Canada; St George's University of London, United Kingdom

## Abstract

**Background:**

Unlike the commonly used method of blue-white screening for gene insertion, a fluorescent protein-based screening method offers a gain-of-function screening process without using any co-factors and a gene fusion product with a fluorescent protein reporter that is further useful in cell imaging studies. However, complications related to protein-folding efficiencies of the gene insert in fusion with fluorescent protein reporters prevent effective on-plate bacterial colony selection leading to its limited use.

**Methodology/Principal Findings:**

Here, we present three methods to tackle this problem. Our first method promotes the folding of the gene insert by using an N-terminal protein such as calmodulin that is well folded and expressed. Under this method, fluorescence was increased more than 30x over control allowing for enhanced screening. Our second method creates a fluorescent protein that is N-terminal to the gene upon insertion, thereby reducing the dependency of the fluorescent protein reporter on the folding of the gene insert. Our third method eliminates any dependence of the fluorescent protein reporter on the folding of the gene insert by using a stop and start sequence for protein translation.

**Conclusions/Significance:**

The three methods together will expand the usefulness of fluorescence on-plate screening and offer a powerful alternative to blue-white screening.

## Introduction

Although the insertion of genes into plasmid vectors is amongst the most routinely performed procedures in molecular biology alongside PCR (polymerase chain reaction), the methods for the screening of successful gene insertion remains a tedious process that often involves running gel electrophoresis on restriction digestions[1] or PCR reactions of many bacterial colonies[2] to check for gene integration. To address this problem, the blue-white colorimetric screen was developed to allow on-plate screening of plasmid integration[3]. This commonly used on-plate screening method employs an engineered *lacZα* protein with an internal multiple cloning site (MCS). In the presence of the chemical X-gal, β-galactosidase activity is detected via blue bacterial colonies. Insertion of PCR products within the MCS disrupts translation of *lacZα* preventing transformed bacteria from turning blue and thus, allowing for on-plate detection of successful gene integration. Complicating factors such as the spontaneous deletion of the *lacZα* gene during the cloning process and the insertion of genes that do not disrupt *lacZα* function lead to false-positive or false-negative screens, respectively[4], [5]. In contrast to screening for the loss of *lacZα* function, fluorescent protein-based screening is based on a gain of fluorescence that can increase screening fidelity as the fluorescence property cannot be acquired spontaneously. Furthermore, exogenous chemical co-factors such as X-gal are not required and genes fused with fluorescent proteins are often the desired final products for further cell imaging studies.

Fluorescence screening of gene insertion on bacterial culture plates can be performed using the pCfvtx plasmid from Truong's cassette methodology because the successful insertion creates a C-terminal fusion of the gene with Venus (yellow fluorescent protein mutant)[6]. However, fluorescence screening is limited by the folding stability of the inserted gene as proper folding of the downstream fluorescent protein is directly related to the folding robustness of N-terminal proteins[7], [8]. Furthermore, since gene cloning is performed in *e. coli* which may lack required chaperone proteins, the translated protein may mis-fold or aggregate in the host organism[9]. These factors adversely affect the folding of the fluorescent protein, hindering successful screens using fluorescence. Here, we address the negative effects of protein folding on fluorescence screening with three alternative methods that utilize fluorescent proteins to screen for successful gene integration.

## Results and Discussion

**Figure 1:**
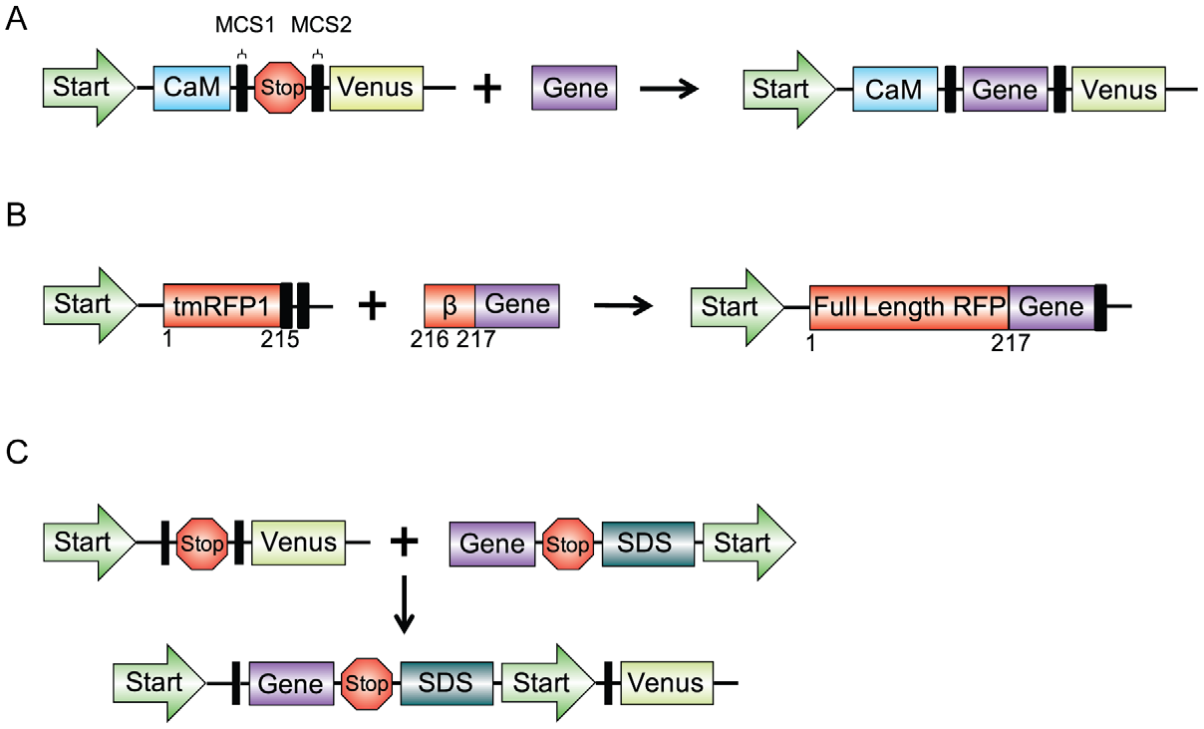
Vector constructs of the three alternative methods of screening for gene insertion using fluorescent proteins. **A**) N-terminally situated well-folded and expressed protein (CaM) to facilitate folding of downstream insert and reporter proteins. **B**) Non-fluorescent C-terminally truncated fluorescent protein (tmRFP1) being completed with gene insert (β) resulting in fluorescence of the fluorescent protein. **C**) Gene insert containing a stop codon, Shine-Dalgarno sequence (SDS), and initiation start codon.

**Figure 2:**
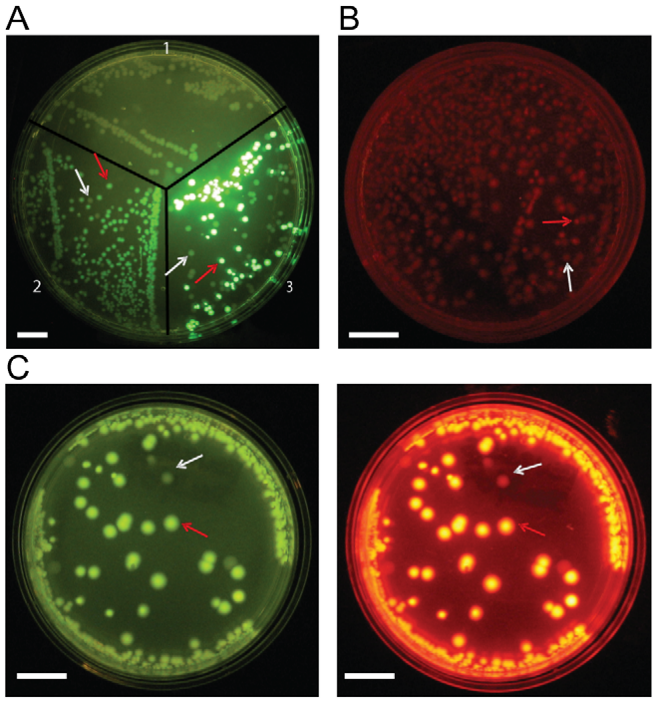
Fluorescent images of bacterial platings using the three alternative methods to insert a gene fragment. **A**) Three different constructs were expressed in *e.coli* and plated to compare on-plate YFP fluorescent intensities. The gene encoding the fluorescent protein hcRed was cloned into the constructs in quadrant (QD) 2 and 3. QD1 – *E.coli* with no fluorescent proteins (control); QD2 – pCfvtx construct; QD3 – Construct with N-terminally situated CaM. White arrows represent non-fluorescent colonies; Red arrows indicate fluorescent colonies. **B**) Truncated mRFP1 was completed upon gene insert and was able to show fluorescence. **C**) Bacterial platings showing both YFP and RFP fluorescence indicating that gene was successfully inserted. Note that YFP fluorescence of successful colonies are strong enough to easily differentiate it from non-fluorescent colonies. Scale bar, 1 cm.

One method to enhance fluorescence screening is to promote the folding or enhanced expression of the gene insert by an N-terminal protein that is well-folded and expressed such as calmodulin (CaM). A poorly folded protein negatively affects folding of downstream proteins. However, a well-folded protein might facilitate increased protein expression levels by overcoming translation initiation. This results in the production of increased downstream fluorescent proteins allowing for improved fluorescence detection. In other studies, we have noticed that fusion protein containing CaM and Venus showed very bright fluorescence, suggesting that CaM is particularly well-folded. Thus, we constructed our plasmid expression vector with CaM situated N-terminal to the MCS1 (Fig. 1A). Testing of this plasmid vector for enhanced fluorescence screening was conducted by inserting the PCR fragment encoding the fluorescent protein hcRed (between MCS1 and MCS2) [10]. Although it is not strictly necessary for the inserted gene to be fluorescent as gene insertion into our plasmid expression vector will induce Venus fluorescence for screening, hcRed was chosen because it also expresses fluorescence and can be used as a simple additional marker for gene insertion. When transformed colonies were plated on agar plates and incubated overnight at 37°C, a substantial increase in Venus fluorescence intensity was observed relative to the standard insertion into the pCfvtx plasmid under a fluorescence plate reader (Fig. 2A). Enhanced fluorescence screening was also seen when other gene of varying length and folding efficiency was inserted into the N-terminally situated CaM plasmid expression vector (Fig. S1). To quantify the intensities of the Venus fluorescence, fluorescent colonies of both types were picked and grown overnight in LB broth. Bacterial culture growths were then adjusted to obtain approximately equal cell densities as measured by OD600 and fluorescence intensities were measured with a fluorometer. The expression vector with CaM showed 30.2x increased in fluorescence relative to the control whereas the standard insertion into the pCfvtx plasmid only showed a 3.2x increase over control (Fig. S2). To confirm that hcRed DNA fragments had been inserted, plasmids were extracted from fluorescent colonies and subjected to electrophoresis after restriction enzyme digestion. The effectiveness of this particular expression plasmid vector in discriminating bacterial colonies with successfully inserted gene fragments from unsuccessful colonies was conducted by a serial dilution of the hcRed insert to produce sub-optimal ligation reaction (Fig. S3).

**Figure 3:**
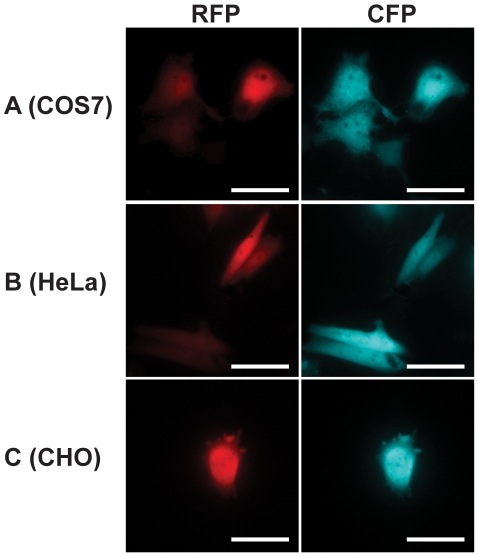
Cell cultures expressing the rescued truncated mRFP1 fused with Cerulean. The plasmid expression vector expressing the rescued truncated mRFP1 fused with Cerulean fluorescent protein directly transfected into three different cell lines. **A**) COS7; **B**) CHO; **C**) HeLa. In all three cell lines, transfected cells expressed both red and cyan fluorescence. Scale bar, 10 µm.

Since the most N-terminal protein has the best opportunity for proper protein-folding and proper folding of our reporter fluorescent protein is imperative for efficient screening, another method to enhance on-plate fluorescence screening is creating gene inserts such that its integration will reconstitute a truncated N-terminal fluorescent protein. In this method, a reporter fluorescent protein was positioned N-terminal of the gene insert so that it could efficiently report the insertion of a desirable gene into the plasmid. Since fluorescence of the fluorescent protein depends on the proper formation of the β-barrel structure[11], [12], [13], fluorescence can be abolished by truncating the fluorescent protein at the C-terminus and disrupting this β-barrel structure[14]. Then, by designing PCR fragments to include the last beta strand of the fluorescent protein, the inserted PCR product will complete the truncated fluorescent protein and restore fluorescence (Fig. 1B). It is important to note however, that there is a possibility that the downstream protein could be inserted with a frame shift after the rescuing last beta strand[15]. This would result in the upstream fluorescent protein gaining the missing strand and expressing fluorescence however, the downstream protein would be out of frame and thus not expressed. Going back to our method, mRFP1 was chosen as the fluorescent protein for truncation as it is stable and matures quickly[16]. Successive C-terminal truncation of mRFP1 showed that fluorescence was abolished after 10 amino acids were removed (Fig. S4, S5). Surprisingly, we found that mRFP1 fluorescence intensity increased after 7 amino acids were eliminated. In order to reduce cost and increase PCR efficiency, the ideal complementary strand to the truncated mRFP1 should be as short as possible while still allowing for fluorescence. Thus, PCR primers for Cerulean[17] (cyan fluorescent protein mutant) were designed to methodically complete the truncated mRFP1. Again, Cerulean was chosen as it acts as an additional marker for gene insertion as well as providing a simple assay for detecting the presence of any frame shifts after the rescuing last beta strand as any frame shifts would abrogate Cerulean fluorescence. When these PCR fragments were inserted, we found that the addition of the amino acids RA to the truncated mRFP1 yielded the brightest fluorescent colonies (Fig. 2B). To confirm PCR fragments were successfully inserted, extracted plasmids from fluorescent colonies were sequenced. In addition, because the expression vector backbone contains a dual promoter system for expression in bacterial and mammalian cells, the final construct can be used directly in cell imaging studies. As a demonstration, the rescued truncated mRFP1 fused with Cerulean was directly transfected into three different cell lines (Fig. 3).

To eliminate the dependence of fluorescence screens on the protein-folding efficiency of the fusion proteins, another method is to control the separate translation of the reporter fluorescent protein using a sequence containing a stop codon, Shine-Dalgarno sequence[18] and a start codon. This sequence was incorporated at the 3′- PCR primer sequence in the respective order (Fig. 1C and Fig. S6). When this PCR fragment was inserted, the stop codon stops the translation of the inserted gene while the Shine-Dalgarno sequence and start codon begin the translation of the reporter fluorescent protein. This separates the expression of the reporter protein from any folding influences of the inserted gene. Using this method, mRFP1 was cloned into the original pCfvtx plasmid. On-plate colonies showed Venus fluorescence as well as red fluorescence, indicative of successful gene insertion (Fig. 2C). To validate that this method produces two separate proteins, proteins were extracted from a fluorescent colony and fluorescent proteins were separated by SDS-PAGE (Fig. S7).

While fluorescence on-plate screening has been available for many years, its adoption has lagged as it depends extensively on the folding efficiency of the gene insert in fusion with the reporter fluorescent protein. Here, we developed three methods to address this shortcoming. By promoting the better folding and expression of the gene insert using CaM, our first method substantially enhanced the fluorescence allowing for clear selection of successful colonies. By completing an N-terminal truncated fluorescent protein with our gene insert, our second method alternatively reduced fluorescence dependence on the folding of the gene insert by allowing our reporter fluorescent protein to fold first. Finally, by using a sequence containing a stop codon, Shine-Dalgarno sequence, and start codon, our third method alternatively eliminates the folding effect of the gene insert because the fluorescent protein is expressed separately from the gene insert. While the third method can be used as the general case, the first and second methods are complementary as the desired final products are often fusions with fluorescent proteins.

## Materials and Methods

### DNA Construction and Testing

All constructs were subcloned using the pCfvtx vector as described previously [6]. For the vector construct containing CaM, a PCR product encoding the CaM (Human) was digested using *Nco*I and *Spe*I, and then inserted at the N-terminal end of the MCS1 of pCfvtx. To test this construct, a PCR product encoding hcRed was digested with *BaM*HI and *Nhe*I and inserted between MCS1 and MCS2. For the vector construct containing the truncated mRFP1, various C-terminal deletion mutants of mRFP1 were generated by PCR with primers shown in **Table S1**. These PCR products were digested with *Nco*I and *Nhe*I and inserted between MCS1 and MCS2 of pCfvtx and screened by fluorescence. The YFP (Venus) reporting protein was removed by enzyme digestion with *Pme*I and then ligated. All mutant variants of mRFP1 were checked by DNA sequencing using the forward TriExUp primer (#70846-3; Novagen). To determine the minimal sequence to reconstitute the truncated mRFP1, PCR products of Cerulean (CFP) that also contained the mRFP1 completion sequences were generated with primers shown in **Table S2**. These PCR products were digested with *Nhe*I and *Xho*I and inserted in MCS2. For the last method, the pCfvtx was used as the host vector. Reverse primers containing a stop codon, Shine-Dalgarno sequence, and start codon (**Fig. S6**), in the respective order, were used to create PCR products that encoded mRFP1. These PCR products were enzyme digested with *Nco*I and *Nhe*I and inserted between MCS1 and MCS2.

### Fluorescent Plate Screening

Plates were screened using a fluorescence plate reader (Montreal Biotech Inc.). Venus was excited and observed with a 488±20 nm bandpass filter and 525 nm longpass filter, respectively; mRFP1 was excited and observed with a 540±20 nm bandpass filter and 580 nm longpass filter respectively.

### Cell Density and Fluorometer Readings

For cell density readings, suspended cells were pelleted and growth media were removed. Cells were washed and re-suspended with Tris Buffer (50 mM TRIS +100 mM NaCl; pH 7.5). Cell densities were measured at OD600 using the BioPhotometer (Eppendorf) and diluted with Tris buffer until readings were approximately equal. Fluorescence readings were taken by exciting samples at 440 nm and emission intensities were obtained by scanning sample emissions from 460 nm–620 nm using a fluorometer (Perkin Elmer, LS50B).

### Bacterial Platings

Plasmids were transformed into DH5α competent *E.coli* cells (Invitrogen) and transferred to 1 mL LB broth with ampicillin for 3 h at 37°C. 60 µL were plated on agar (with ampicillin) plates and left in incubator overnight at 37°C. All photos were taken with a Canon digital camera (PowerShot A530).

### Preparation of SDS-PAGE

To prepare samples for SDS-PAGE, fluorescent colonies were picked and grown in 4 mL of LB broth overnight at 37°C. Cells were obtained by pelleting culture growth and washing and re-suspending pellet with 350 µL of Tris buffer at 4°C. Cells were then lysed by sonication (Branson Sonifier 250) on ice for 1 minute at 30% duty cycle. Cell lysate was extracted after centrifugation for 3 minutes at 13,200 rpm. Fluorescence SDS-PAGE [19] was performed using NuPage Novex 4–12% Bis-Tris Pre-cast Gel (Invitrogen) and accompanying protocols.

### Cell Culture and Transfection of Cell Lines

Cell lines (COS7, CHO, HeLa) were maintained in Dulbecco's Modified Eagle's Medium supplemented with 25 mM D-glucose, 1 mM sodium pyruvate, 4 mM L-glutamine (Invitrogen), 10% fetal bovine serum, and 10 ml/L Penicillin-Streptomycin solution (Sigma-Aldrich) in separate T5 flasks. At 95% confluency, cells were passaged using 0.05% trypsin with EDTA (Sigma-Aldrich) and seeded onto glass-bottom dishes (MatTek Corp.) at 1∶10 dilution. Plasmid DNA transfection of cell lines was conducted using Lipofectamine 2000 according to manufacturer's protocols (Invitrogen).

## Supporting Information

Figure S1 Comparison of fluorescence of other gene inserts. Two additional genes of varying length and folding efficiency were inserted into the N-terminally situated CaM plasmid expression vector. A) Gene encoding RhoA, ∼330 a.a. in length, show enhanced fluorescence when cloned into the CaM plasmid expression vector, quadrant (QD) 2, relative to the control pCfvtx plasmid, QD1. B) Similarly, gene encoding human p21, a ∼200 a.a. protein of poor expression in e.coli, resulted in fluorescent bacterial colonies when cloned into the CaM plasmid expression vector, QD2, while the control pCfvtx plasmid did not express fluorescence, QD1. Scale bar, 1 cm.
(8.44 MB TIF)

Figure S2 Relative intensities of reporting YFP fluorescence. Emission spectrum of YFP fluorescence of construct with N-terminally well-folded and expressed protein (grey line), the pCfvtx plasmid (light grey line), and a non-fluorescent plasmid (black line) showing varying intensities.
(2.32 MB TIF)

Figure S3 Platings of titration of sub-optimal ligations. Platings of sub-optimal ligation reactions of serial diluted inserts of hcRed gene fragment into N-situated CaM plasmid expression vector. Efficiencies: A) 100% (positive control); B) 50%; C) 25%; D) 12.5%; E) 6.25%; F) 3.125%; G) 1%; H) 0% (negative control). Scale bar, 1 cm.
(9.59 MB TIF)

Figure S4 C-terminal truncation of mRFP1. Schematic diagram of the truncation of the last beta strand (C-terminus) of mRFP1.
(7.31 MB TIF)

Figure S5 Relative fluorescence of truncated mRFP1. Fluorescence image of pelleted e.coli cells of the truncated mRFP1 showing fluorescence abrogated after 10 amino acids were removed. Of note is that fluorescence intensity increased when 7 amino acids were removed. Refer to Table S2 for description of t1 (tRFP1Ceru), t2 (tRFP2Ceru), and t3 (tRFP3Ceru). Non-fluorescent pelleted e.coli cells were used as control.
(4.70 MB TIF)

Figure S6 General reverse primer sequence. Schematic diagram of general reverse primer containing a stop codon, Shine-Dalgarno sequence (RBS), and initiation codon. ‘X’ represents arbitrary nucleotides. For more certainty of stopping read-though, add another stop codon that is out of frame.
(4.69 MB TIF)

Figure S7 SDS-PAGE separation of fluorescent proteins. Fluorescence image of separated fluorescent proteins by SDS-PAGE. Lane 1 - Venus (Control); Lane 2 - mRFP1 (Control); Lane 3 - mRFP1-stop-SDS-start-venus; Lane 4 - mRFP1-venus fusion.
(5.95 MB TIF)

Table S1 The forward primer 5′- CATGCCATGGGCGCCTCCTCCGAGGACGTCATC -3′ was common in all cases. Underlined sequences are NheI restriction sites except for the forward primer which is NcoI.
(0.03 MB DOC)

Table S2 The reverse primer 5′- CCCTCGAGACTAGCGGCGGCGGTCACGAA -3′ was common in all cases. The underlined sequences are NheI restriction sites except in the reverse primer were it is XhoI. The bold sequences represent the amino acids to complete the truncated mRFP1.
(0.03 MB DOC)
