# Supplementary figures and images for: Fluorescent Protein-Based Methods for On-Plate Screening of Gene Insertion
Source: PLoS One. 2010 Dec 10;5(12):e14274. doi: 10.1371/journal.pone.0014274 (PMC3000809; doi:10.1371/journal.pone.0014274)

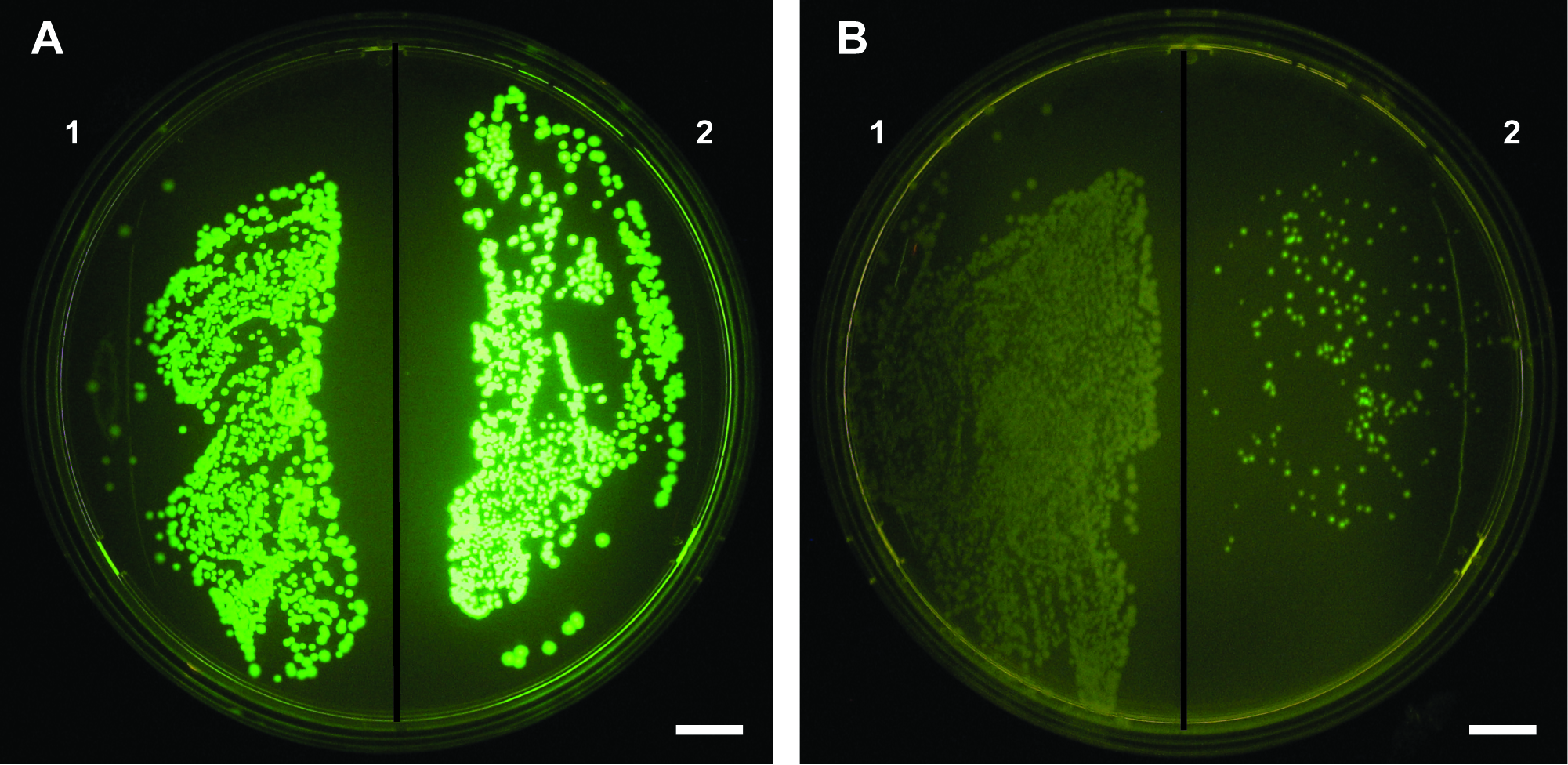

Supplement: Figure S1 — Comparison of fluorescence of other gene inserts. Two additional genes of varying length and folding efficiency were inserted into the N-terminally situated CaM plasmid expression vector. A) Gene encoding RhoA, ∼330 a.a. in length, show enhanced fluorescence when cloned into the CaM plasmid expression vector, quadrant (QD) 2, relative to the control pCfvtx plasmid, QD1. B) Similarly, gene encoding human p21, a ∼200 a.a. protein of poor expression in e.coli, resulted in fluorescent bacterial colonies when cloned into the CaM plasmid expression vector, QD2, while the control pCfvtx plasmid did not express fluorescence, QD1. Scale bar, 1 cm. (8.44 MB TIF) [file pone.0014274.s001.tif]

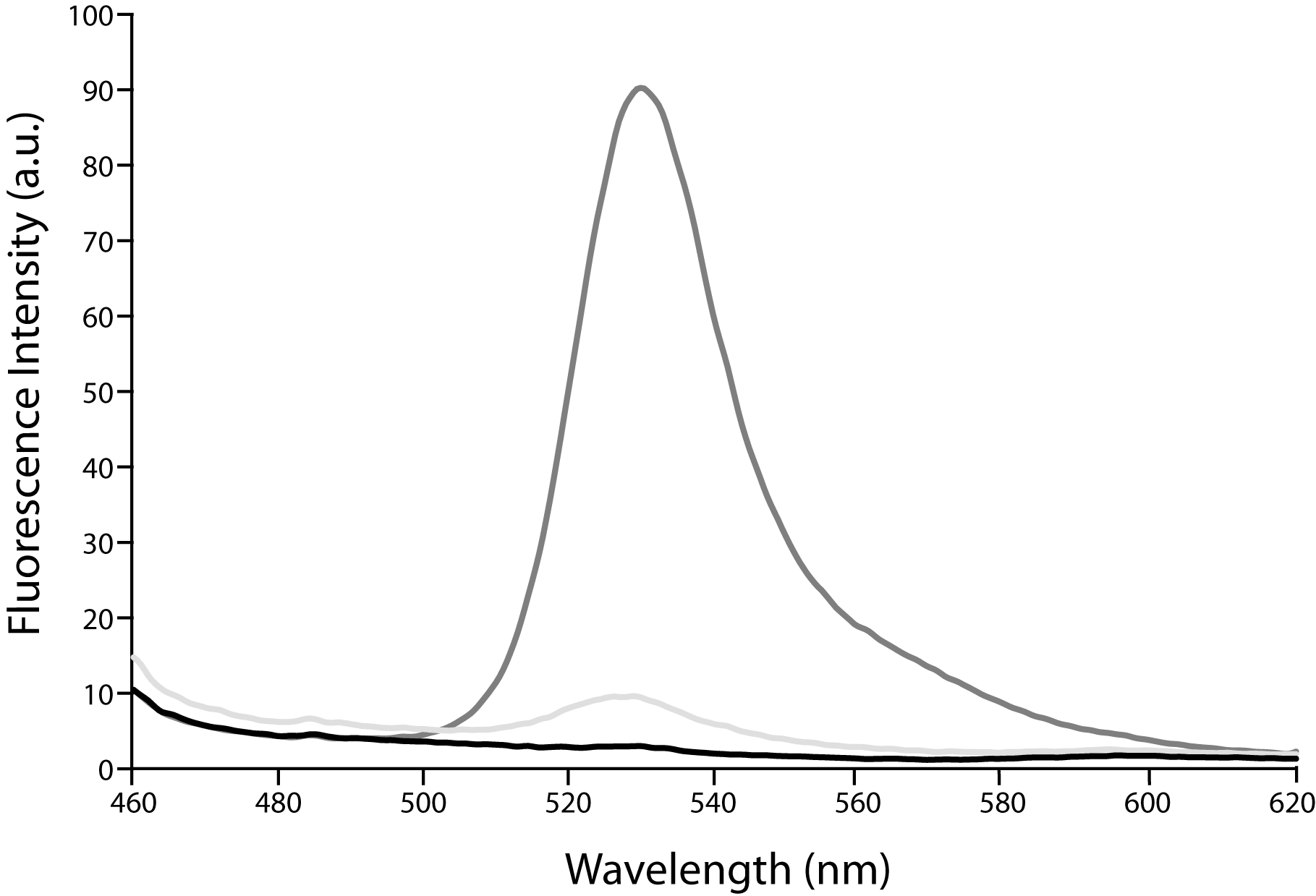

Supplement: Figure S2 — Relative intensities of reporting YFP fluorescence. Emission spectrum of YFP fluorescence of construct with N-terminally well-folded and expressed protein (grey line), the pCfvtx plasmid (light grey line), and a non-fluorescent plasmid (black line) showing varying intensities. (2.32 MB TIF) [file pone.0014274.s002.tif]

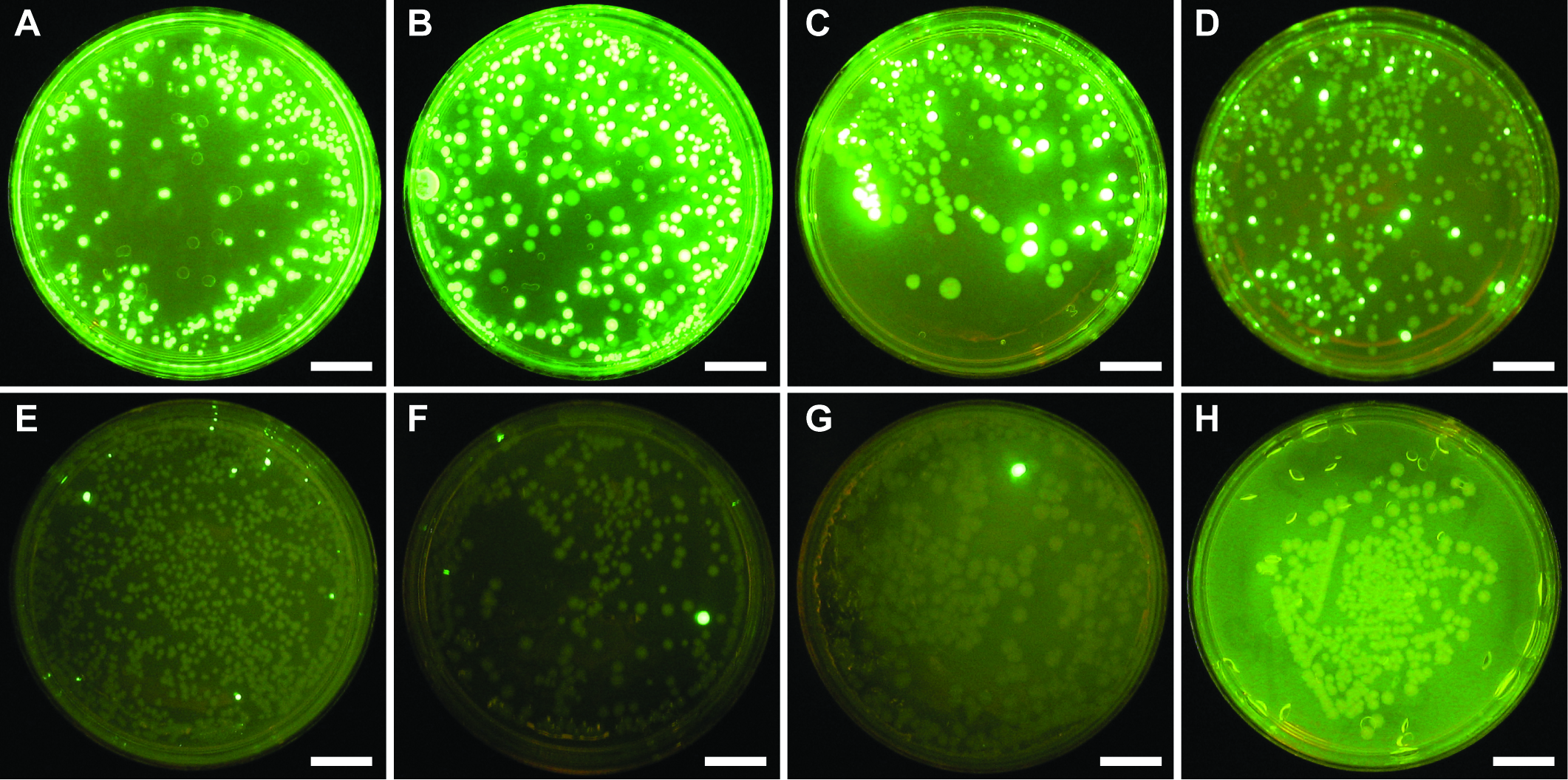

Supplement: Figure S3 — Platings of titration of sub-optimal ligations. Platings of sub-optimal ligation reactions of serial diluted inserts of hcRed gene fragment into N-situated CaM plasmid expression vector. Efficiencies: A) 100% (positive control); B) 50%; C) 25%; D) 12.5%; E) 6.25%; F) 3.125%; G) 1%; H) 0% (negative control). Scale bar, 1 cm. (9.59 MB TIF) [file pone.0014274.s003.tif]

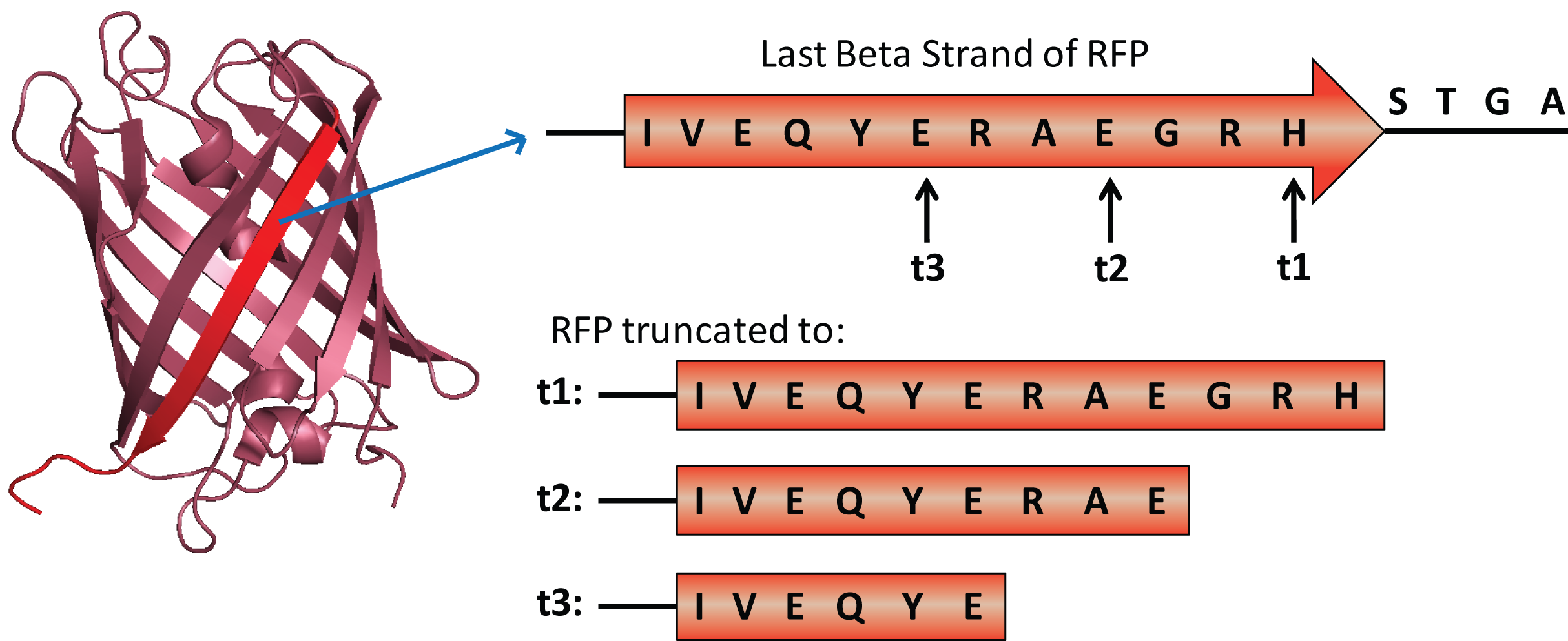

Supplement: Figure S4 — C-terminal truncation of mRFP1. Schematic diagram of the truncation of the last beta strand (C-terminus) of mRFP1. (7.31 MB TIF) [file pone.0014274.s004.tif]

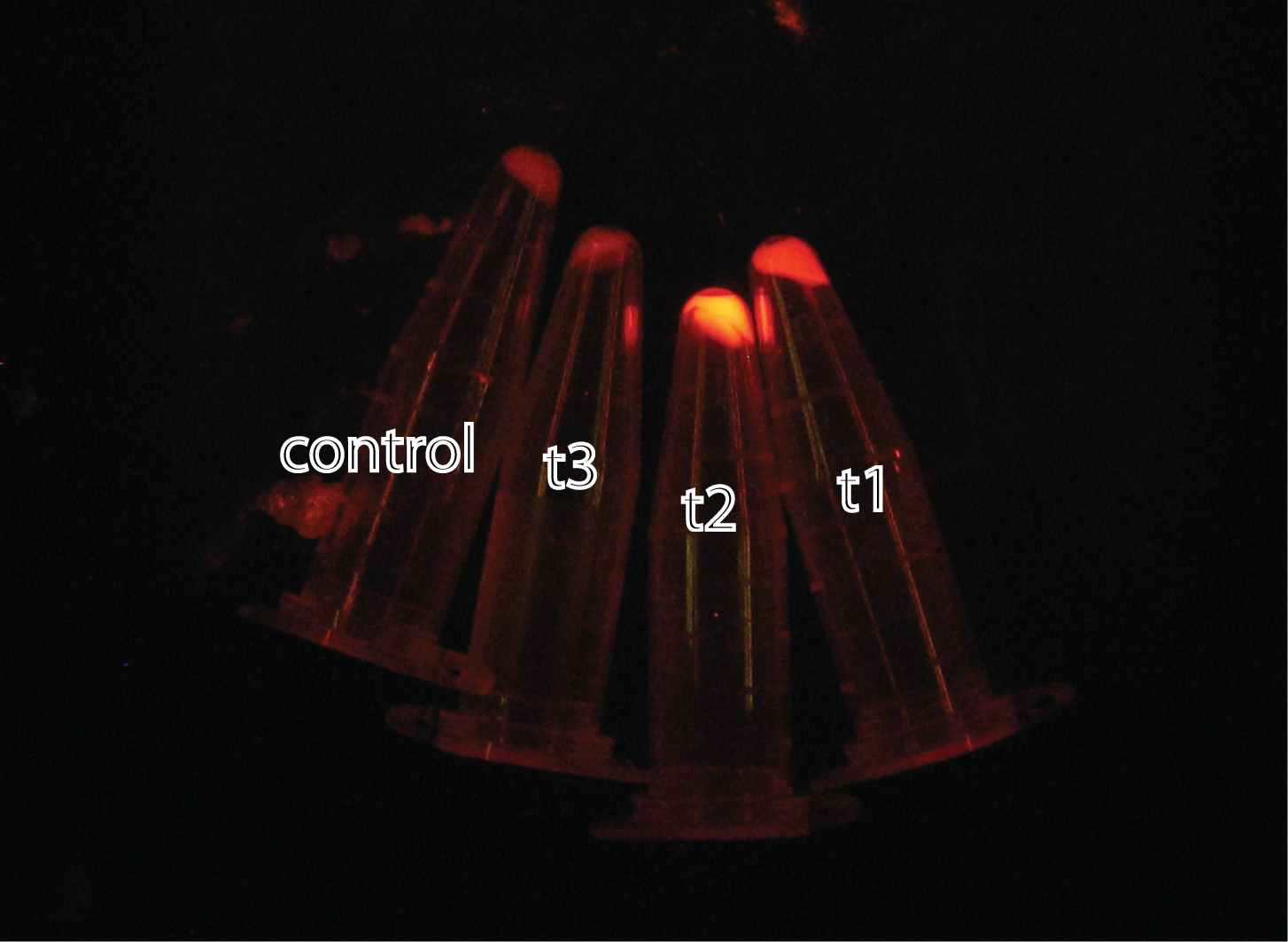

Supplement: Figure S5 — Relative fluorescence of truncated mRFP1. Fluorescence image of pelleted e.coli cells of the truncated mRFP1 showing fluorescence abrogated after 10 amino acids were removed. Of note is that fluorescence intensity increased when 7 amino acids were removed. Refer to Table S2 for description of t1 (tRFP1Ceru), t2 (tRFP2Ceru), and t3 (tRFP3Ceru). Non-fluorescent pelleted e.coli cells were used as control. (4.70 MB TIF) [file pone.0014274.s005.tif]

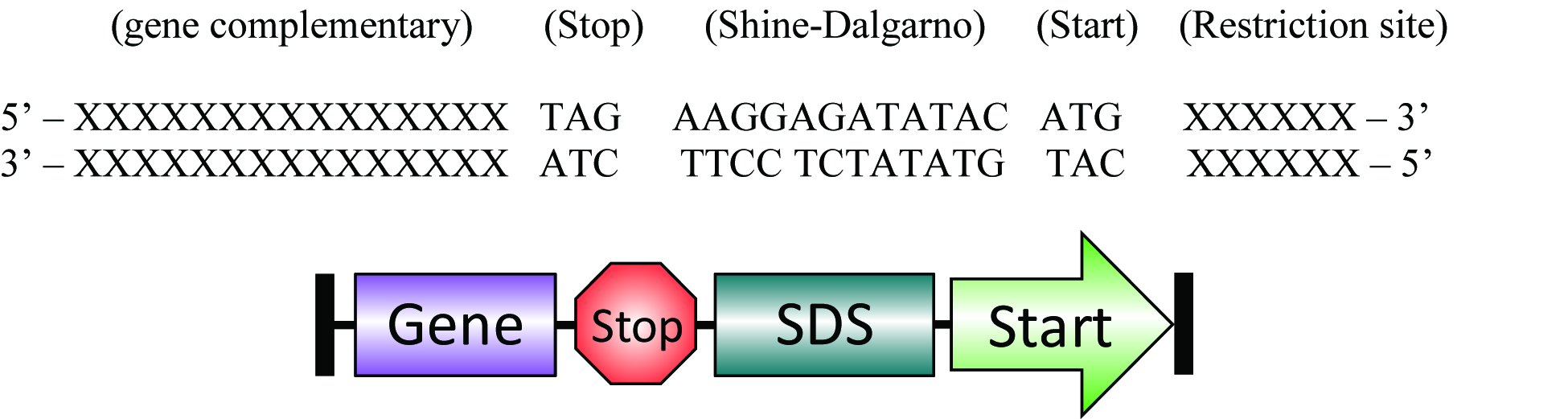

Supplement: Figure S6 — General reverse primer sequence. Schematic diagram of general reverse primer containing a stop codon, Shine-Dalgarno sequence (RBS), and initiation codon. ‘X’ represents arbitrary nucleotides. For more certainty of stopping read-though, add another stop codon that is out of frame. (4.69 MB TIF) [file pone.0014274.s006.tif]

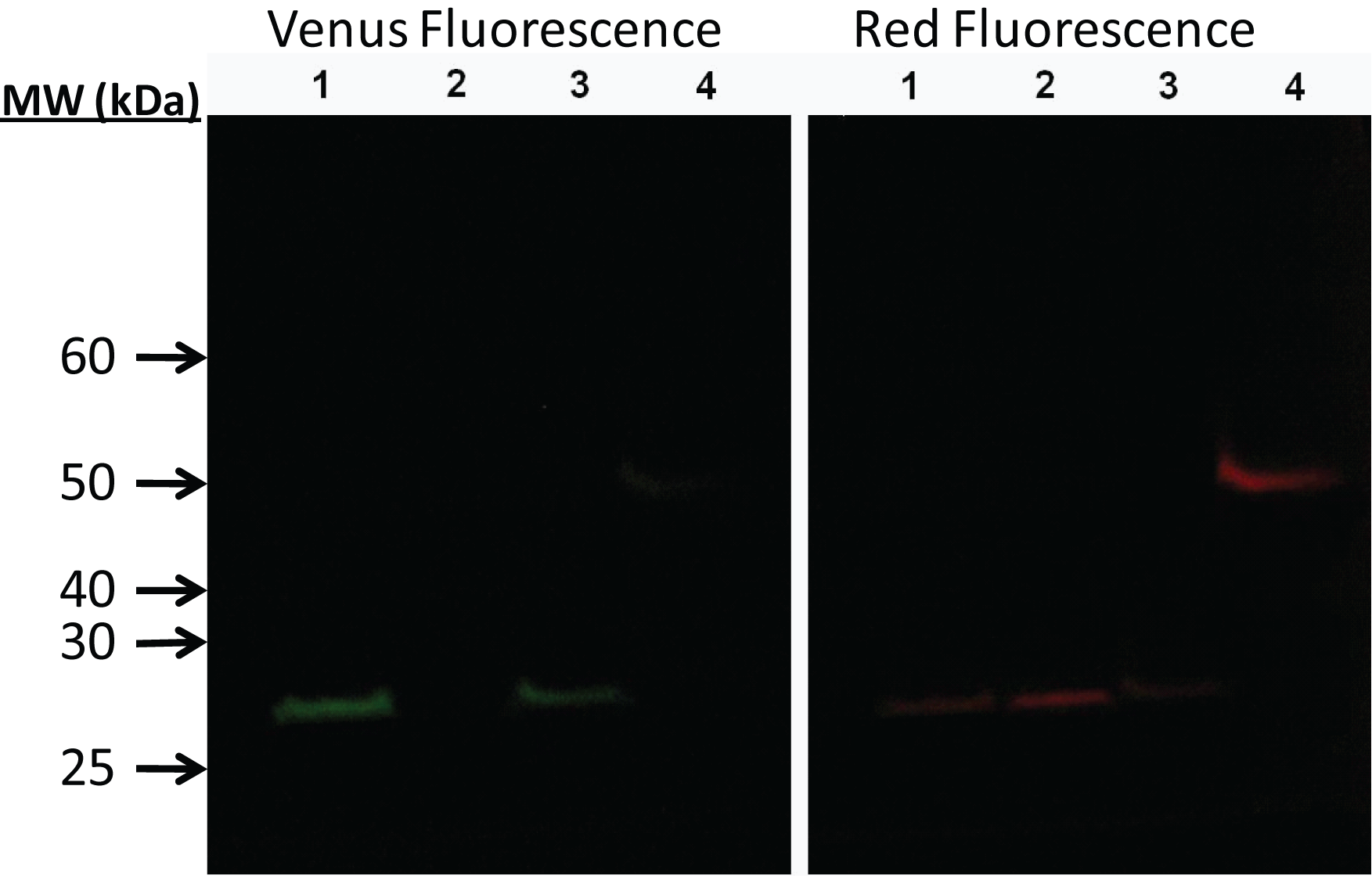

Supplement: Figure S7 — SDS-PAGE separation of fluorescent proteins. Fluorescence image of separated fluorescent proteins by SDS-PAGE. Lane 1 - Venus (Control); Lane 2 - mRFP1 (Control); Lane 3 - mRFP1-stop-SDS-start-venus; Lane 4 - mRFP1-venus fusion. (5.95 MB TIF) [file pone.0014274.s007.tif]
