# Supplementary material for: Fluorescent Protein-Based Methods for On-Plate Screening of Gene Insertion
Source: PLoS One. 2010 Dec 10;5(12):e14274. doi: 10.1371/journal.pone.0014274 (PMC3000809; doi:10.1371/journal.pone.0014274)
Supplement: Table S1 — The forward primer 5′- CATGCCATGGGCGCCTCCTCCGAGGACGTCATC -3′ was common in all cases. Underlined sequences are NheI restriction sites except for the forward primer which is NcoI. (0.03 MB DOC) [file pone.0014274.s008.doc]

**Table S1** Reverse primers for truncated mRFP1

| Name | Sequence of Reverse Primers |
| --- | --- |
| t1 | 5’ –CGCGCGGCTCCCGGCGGTGCGATCGGC - 3’ |
| t2 | 5’ – CTTGTCATGCTCGCGCGGCTCCGATCGGC - 3’ |
| t3 | 5’ – TGTGGTAGCACCTTGTCATGCTCCGATCGGC - 3’ |
