# Supplementary material for: Fluorescent Protein-Based Methods for On-Plate Screening of Gene Insertion
Source: PLoS One. 2010 Dec 10;5(12):e14274. doi: 10.1371/journal.pone.0014274 (PMC3000809; doi:10.1371/journal.pone.0014274)
Supplement: Table S2 — The reverse primer 5′- CCCTCGAGACTAGCGGCGGCGGTCACGAA -3′ was common in all cases. The underlined sequences are NheI restriction sites except in the reverse primer were it is XhoI. The bold sequences represent the amino acids to complete the truncated mRFP1. (0.03 MB DOC) [file pone.0014274.s009.doc]

**Table S2** Forward primers for truncated mRFP1 completion

| Name | Sequence of Forward Primers |
| --- | --- |
| tRFP1Ceru (RAE) | 5’ - CGGCTAGC**CGCGCCGAG**agcaagggcgaggagctgttca - 3’ |
| tRFP2Ceru (RA) | 5’ - CGGCTAGC**CGCGCC**agcaagggcgaggagctgttca - 3’ |
| tRFP3Ceru (R) | 5’ - CGGCTAGC**CGC**agcaagggcgaggagctgttca - 3’ |
